# Residue 188 of the Hexon protein governs FAdV-4 pathogenicity by activating PINK1/Parkin-mediated mitophagy

**DOI:** 10.1186/s13567-026-01791-1

**Published:** 2026-06-13

**Authors:** Baiyu Wang, Qilong Qiao, Panpan Yang, Minghe Xu, Luyao Qiu, Yutao Zhu, Mengjia Xiang, Yanfang Cong, Dongdong Yang, Jianli Li, Kexiang Yu, Jun Zhao

**Affiliations:** 1 College of Veterinary Medicine, Henan Agricultural University, Zhengzhou, 450046 Henan Province China; 2 Institute of Poultry Science, Shandong Academy of Agricultural Sciences, Jinan, 250100 Shandong Province China

**Keywords:** Fowl adenovirus serotype 4, mitochondrial damage, mitophagy, pathogenicity, viral pathogenesis

## Abstract

Fowl adenovirus serotype 4 (FAdV-4) infection causes significant economic losses to the global poultry industry. Viruses often hijack host cellular machinery to facilitate their replication; however, the mechanisms by which FAdV-4 manipulates host pathways remain poorly defined. Mitochondria, the central hubs for energy metabolism and innate immunity in hepatocytes and cardiomyocytes, are critical targets for viral manipulation, yet their role in FAdV-4 pathogenesis remains unexplored. Here, we demonstrated that FAdV-4 infection caused direct mitochondrial damage and induced PINK1/Parkin-dependent mitophagy both in vitro and in vivo. Moreover, the virus actively hijacked the PINK1/Parkin-mediated mitophagy to enhance viral replication in LMH cells. Inhibition of mitophagy led to an average tenfold reduction in viral replication of pathogenic FAdV-4 in LMH cells (*p* < 0.05). Strikingly, residue 188 in the Hexon protein, a key virulence determinant, differentially regulated mitophagy: the R188I mutation in the pathogenic FAdV-4 attenuated mitophagy, whereas the I188R mutation in nonpathogenic FAdV-4 enhanced this process. This study elucidated the viral exploitation of mitophagy by FAdV-4 to promote viral replication, and established Hexon residue 188 and the mitophagy pathway as prime targets for developing novel therapeutics against avian adenoviral diseases.

## Introduction

Hepatitis-hydropericardium syndrome (HHS) induced by hypervirulent fowl adenovirus serotype 4 (FAdV-4) is a widely transmitted infectious disease in poultry, causing significant economic losses to poultry farms in affected regions, including parts of Asia, the Americas, and Europe [1, 2]. The disease mainly affects chickens from 3 to 6 weeks of age, resulting in severe hepatitis, accumulation of fluid in the pericardial sac and acute mortality [3–5]. FAdV-4 is an icosahedral, nonenveloped, double-stranded DNA virus. The viral genome is tightly packed and surrounded by the viral capsid composed of three major structural proteins, Hexon, Fiber, and Penton. The trapezoidal, cylindrical-shaped Hexon homotrimer is the main capsid protein [6, 7]. While previous studies have established the pivotal role of Hexon residue 188 in determining viral pathogenicity [8–11], the exact cellular mechanisms through which this molecular determinant affects pathogenesis have yet to be fully elucidated.

The pathophysiological significance of mitochondrial integrity in FAdV-4-targeted organs warrants particular attention. In cardiomyocytes, mitochondria constitute 30–40% of cellular volume, forming intricate networks that regulate not only oxidative phosphorylation for ATP synthesis but also calcium homeostasis, reactive oxygen species (ROS) signaling, and apoptotic cascade initiation. These energy-demanding cells maintain strict mitochondrial quality control and homeostasis through dynamic fusion/fission cycles and selective autophagy—processes particularly vulnerable to viral infection [12]. Similarly, mitochondria in hepatocytes are essential for xenobiotic detoxification, β-oxidation of fatty acids, and gluconeogenesis. The liver’s unique position as both metabolic hub and immune sentinel renders its mitochondrial network a critical battlefield during viral pathogenesis [13]. Evidence has shown that DNA viruses frequently target mitochondrial dynamics to manipulate host cell survival decisions [14]. Mitochondria exhibiting irreversible damage initiate a molecular cascade that facilitates their encapsulation into autophagosomes. These mitochondrion-containing vesicles subsequently fuse with lysosomes, resulting in the enzymatic breakdown of mitochondrial fragments into fundamental biomolecules for cellular reutilization. This process is termed mitophagy, a selective autophagic clearance of damaged mitochondria that involves over 40 autophagy-related proteins (ATGs). Mitophagy presents as a double-edged sword in viral infections: while eliminating compromised organelles could restrict viral replication, certain pathogens exploit this pathway to evade immune detection or acquire metabolic advantages [13, 15].

The phosphatase and tensin homolog (PTEN)-induced putative kinase protein 1 (PINK1)/Parkin-regulated pathway is the best characterized mitophagy pathway for mitochondrial quality control. PINK1 is a mitochondrial serine/threonine kinase acting as a stress sensor. Under normal conditions, PINK1 is imported into the mitochondrial inner membrane and cleaved by presenilin-associated rhomboid-like (PARL) protease. However, under stressed conditions, PINK1 recruits cytosolic Parkin, an E3 ubiquitin ligase that ubiquitinates the outer mitochondrial membrane proteins, marking mitochondria for degradation. The ubiquitinated substrates bind to autophagy adaptors, such as P62/SQSTM1, which link to LC3 (microtubule-associated protein 1 light chain 3) on the autophagosomal membranes, facilitating engulfment and lysosomal clearance of the damaged mitochondria [16, 17].

Emerging evidence suggests FAdV-4 is capable of inducing inflammation and autophagic activity in hosts and may hijack cellular autophagy to promote viral replication [18–22]. However, the interplay between FAdV-4 infection, mitochondrial dynamics, and mitophagy remains unexplored. Whether and how Hexon residue 188 modulates mitochondrial quality control during FAdV-4 infection remains unknown. The present study assesses mitochondrial injury in chicken hepatocytes and cardiomyocytes and PINK1/Parkin-regulated mitophagy induced by FAdV-4 of varying virulence. Addressing these gaps will deepen our understanding of fowl adenovirus pathogenesis and inform therapeutic strategies targeting mitochondrial quality control.

## Materials and methods

### Cell, viruses and antibodies

The hypervirulent strain of FAdV-4, identified as CH/HNJZ/2015 and referred to as HNJZ (GenBank accession no. KU558760), was isolated by our lab in 2015 from chickens with HHS in Henan Province, China, as previously reported [10, 23]. The nonpathogenic FAdV-4 strain ON1 (GenBank accession no. GU188428) was generously supplied by Dr Éva Nagy from the Department of Pathobiology at the University of Guelph in Canada. The recombinant viruses H/H/R188I and O/O/I188R were previously constructed in our lab. H/H/R188I was constructed by substitution of arginine at position 188 of Hexon in the hypervirulent strain HNJZ with isoleucine at position 188 of Hexon in the nonpathogenic strain ON1, and O/O/I188R was constructed vice versa [6]. H/H/R188I was characterized as a low-pathogenicity strain whereas O/O/I188R was determined to be pathogenic according to our previous experiments [6]. Virus propagation and cell experiments were conducted on Leghorn male hepatocellular carcinoma (LMH) cells (ATCC, CRL-2117) cultured in DMEM/F12 medium (Thermo Fisher Scientific, USA) supplemented with 10% fetal bovine serum (FBS, AusgeneX, Australia) at 37 °C in a 5% CO_2_ incubator.

The protein expression levels of LC3, P62, Parkin, TOM20, and β-actin were examined by either western blot (WB) or immunofluorescence assay (IFA). The antibodies used for these assays are presented in Table 1.

**Table 1:** **Antibodies used in western blot and immunofluorescence assay**

Antibody	Application	Catalog no.	Supplier
Anti-LC3 polyclonal	WB	NB100	Novus biologicals (USA)
Anti-P62 monoclonal	WB	66184	Proteintech (China)
Anti-Parkin polyclonal	WB	A11172	ABclonal (China)
Anti-β-actin monoclonal	WB	sc-47778	Santa cruz biotechnology (USA)
Anti-TOM20 monoclonal	IF	AF1717	Beyotime (China)
HRP-conjugated goat anti-rabbit IgG	WB	ab6721	Abcam (UK)
HRP-conjugated goat anti-mouse IgG	WB	ab205719	Abcam (UK)
Cy3-conjugated goat anti-rabbit IgG	IF	SA00009-2	Proteintech (China)

### Cell culture and sample collection

To validate whether FAdV-4 infection induced mitochondrial damage and mitophagy in hepatocytes in vitro, LMH cells were cultured in 6-well plates at a density of 2 × 10^6^ cells per well and inoculated with HNJZ, ON1, H/H/R188I, and O/O/I188R at a MOI of 0.001, respectively, with one well left as a blank control. At 48 hours post-infection (hpi), mitochondrial morphology was determined by transmission electron microscopy (TEM). At 72 hpi, total RNA was collected for reverse transcription quantitative polymerase chain reaction (RT-qPCR). Total protein was collected at 24, 48, and 72 hpi for western blot analysis.

### Detection of mitochondrial membrane potential

The mitochondrial membrane potential (MMP) changes were detected using Mitochondrial Membrane Potential Assay Kit with JC-1 (Beyotime, C2006, China). In brief, LMH cells were cultured on 35-mm Petri dishes and inoculated with HNJZ, ON1, H/H/R188I, and O/O/I188R at a MOI of 0.001. Cells were harvested at 48 hpi and subjected to JC-1 staining according to the manufacturer’s instructions. Carbonyl cyanide 3-chlorophenylhydrazone (CCCP), which induces complete dissipation of MMP, was employed as the positive control. Thereafter, cells were photographed with a ZEISS confocal microscope and processed with the same imaging parameters.

### Indirect immunofluorescence assay

Indirect immunofluorescence assay was conducted to observe the co-localization of LC3 and the mitochondrial marker TOM20. LMH cells were cultured in 6-well plates at a density of 1 × 10^6^ cells per well and transfected with 2 μg of pEGFP-LC3 plasmid using Lipofectamine 3000. At 24 h post-transfection, cells were infected with FAdV-4 of different virulence (MOI = 0.001). At 48 hpi, the cells were fixed with 4% paraformaldehyde for 30 min and permeabilized with 0.2% Triton X-100. After blocking with 5% bovine serum albumin (BSA) solution, the cells were incubated with anti-TOM20 antibody diluted 1:200 at 37 °C for 1 h, followed by incubation with a secondary Cy3-conjugated goat anti-rabbit antibody. After washing with PBS containing 0.1% Tween 20 (PBST), the nuclei were stained with DAPI for 5 min before confocal microscopic observation.

### Animal experiment

To assess the impact of FAdV-4 infection on mitochondrial integrity in hepatocytes and cardiomyocytes in vivo, a total of 50 3-week-old specific-pathogen-free (SPF) White Leghorn chickens (Beijing Boehringer Ingelheim Vital Biotechnology Co., Ltd., China) were randomly and equally assigned to five groups: HNJZ-, ON1-, H/H/R188I-, O/O/I188R-infected groups, and an uninfected control group (*n* = 10). SPF chickens were housed separately in negative pressure isolators located in different rooms with sufficient space, water and food supplies and taken cared of under the approval of the Animal Care and Use Committee of Henan Agricultural University. The infected groups received an intramuscular inoculation of 2 × 10^5^ TCID_50_ of the corresponding virus, while the control group received the same volume of serum-free medium. At 3 days post-inoculation, two chickens from each group were euthanized for transmission electron microscopy sample preparation. Additionally, heart and liver tissues from three randomly chosen chickens in each group were collected for protein extraction at 48 and 72 hpi. Upon the death of the HNJZ-infected chickens at approximately 72 hpi, the remaining groups were euthanized, and heart and liver tissues of three chickens from each group were collected for RNA extraction and RT-qPCR. Sample collection was performed by an independent researcher who was blinded to the experimental groups to prevent any potential bias. All euthanasia procedures were performed by skilled personnel via cervical dislocation after carbon dioxide inhalation.

### Transmission electron microscopy

To examine the ultrastructure of mitochondria in LMH cells, the culture supernatant was removed, and 2 mL of 2.5% glutaraldehyde solution was added to the cells, followed by fixation on ice for 5 min. The cells were then gently detached from the bottom of wells using a cell scraper. The cell suspension in the fixation solution was transferred to a microcentrifuge tube with a Pasteur pipette. The samples were then centrifuged at 500 *g* for 15 min to pellet the cells. The supernatant was discarded, and fresh fixation solution was added. The cells were fixed at room temperature for 2 h and stored at 4 °C.

The tissue samples for TEM were prepared immediately after euthanizing the chickens. The target organs were rapidly excised using sterile surgical scissors and placed in a Petri dish. TEM fixation solution was added to completely cover the tissue samples. Using a sharp blade, tissue blocks smaller than 1 mm^3^ were excised and immediately transferred to microcentrifuge tubes containing pre-chilled fixation solution. The entire process was completed as quickly as possible, and the samples were stored at 4 °C. TEM was performed at Wuhan Servicebio Technology Co., Ltd.

### Reverse transcription quantitative PCR (RT-qPCR)

To investigate the mRNA expression of mitophagy-related genes in infected LMH cells, as well as in liver and heart tissues, total RNA was extracted with the RNAsimple Total RNA kit (TIANGEN, China). Briefly, cells were washed once with PBS and lysed using 1 mL of RZ lysis buffer. Total RNA was extracted following the manufacturer’s protocol, and RNA concentration and purity were determined using a NanoDrop spectrophotometer. Reverse transcription of RNA was performed using the FastKing RT Kit (TIANGEN, China), and the resulting cDNA was stored at −20 °C for subsequent use. RT-qPCR was performed with primers presented in Table 2. The expression levels of each target gene were normalized to glyceraldehyde-3-phosphate dehydrogenase (GAPDH), and the relative expression levels were calculated using the 2^−ΔΔCt^ method.

**Table 2:** **Primer sequences for quantitative real-time reverse transcription PCR**

Target gene	Forward primer (5′–3′)	Reverse primer (5′–3′)
LC3	AGTGAAGTGTAGCAGGATGA	AAGCCTTGTGAACGAGAT
PINK1	GATGTGGAACATCTCGGCTG	GATGTGGAACATCTCGGCTG
P62	GACCCAGCCAAGACTACCAT	CAGAGGCATGTAGTTTCGGC
Parkin	GTCCAGCAAAGCATCGTTCA	CAACGATGGAAGGATGCTGG
ATG3	TACTTGGCAATGGGCTTCA	CCATCTGCTTGCACCTTTTG
ATG5	AGAGATGTGTGGTTTGGACGC	GCCGAGGAAGGGCTGTATT
ATG4A	CACAGCAGTGCACATTTGCA	CAGAGTCCTGCTGCGTTCCT
ATG4B	CCCCGATGAAAGCTTCCA	GCTCAGCGATGCTCATTCTG
ATG9	CCACCGCTATGAGACAAGGG	GGTAATGATGGGACTCAGAAGC
ATG12	GGGACCCTCTATGAGTGTTTTG	TGCTTGCGATTCATCCCCAT
Beclin-1	CGTATGGCAACCACTCGTATT	TTATTGTCCCAGAAGAACCTCAG
GAPDH	AGAACATCATCCCAGCGT	AGCCTTCACTACCCTCTTG

### Western blot

For cell samples, LMH cells were washed twice with ice‑cold PBS and then lysed on ice for 20 min in RIPA lysis buffer (Beyotime, China) supplemented with 1× protease inhibitor cocktail (Beyotime, China). Cell lysates were collected into microcentrifuge tubes and centrifuged at 12 000 *g* for 10 min at 4 °C. The supernatants were transferred to fresh tubes and stored at −80 °C until use. For tissue samples, approximately 10 mg of cardiac or hepatic tissue from each group (*n* = 3) was homogenized in 500 µL of RIPA lysis buffer on ice using a glass homogenizer. The homogenate was centrifuged at 12 000 *g* for 5 min at 4 °C, and the resulting supernatant was collected for western blot analysis. Protein concentrations were determined using a BCA assay kit (Thermo Fisher Scientific, USA). Samples were separated via sodium dodecyl sulfate-polyacrylamide gel electrophoresis (SDS-PAGE) and transferred to nitrocellulose membranes. After blocking with 5% skimmed milk, membranes were incubated with primary antibodies against Parkin, P62, LC3, and β-actin, followed by incubation with HRP-conjugated secondary antibodies. Signals were developed using Immobilon Western HRP Substrate (Millipore, WBKLS0050) and visualized with an Amersham Imager 600 RGB scanner.

### Pharmacological and genetic inhibition of mitophagy

To investigate the impact of mitophagy on viral replication, LMH cells were treated with the mitophagy inhibitor Cyclosporin A (CsA, MCE, HY-B0579, China) for 24 h. Cell viability was assessed using the CCK-8 assay to determine the optimal noncytotoxic concentrations of CsA (ranging from 1 to 200 μM) for subsequent experiments. On the basis of the viability results, cells were pretreated with a selected concentration of CsA (1 μM) for 6 h prior to infection with FAdV-4 (wild-type or Hexon residue 188 mutant strains) [24]. Viral replication efficiency was evaluated at 48 hpi by qPCR using a previously established standard curve method targeting ORF14 of FAdV-4 [10].

In parallel, LMH cells were transfected with siParkin (sense: CCAGCAAAGCAUCGUUCAUTT; antisense: AUGAACGAUGCUUUGCUGGTT) using Lipofectamine 3000 (Thermo Fisher Scientific, USA) [25]. At 48 h post-transfection, cells were infected with FAdV-4 at a MOI of 0.001 (wild-type or Hexon residue 188 mutant strains). Viral replication efficiency was evaluated at 48 hpi by qPCR.

### Statistical analysis

Grayscale analysis of western blot and average fluorescent signal intensity calculation were performed with ImageJ software. Quantitative results are presented as mean ± standard error of the mean. Comparisons of MMP, western blot, viral load, mitophagy-related gene expression data were performed using unpaired Student’s *t*-test or one-way ANOVA. The normality of data was confirmed by the D’Agostino–Pearson normality test. All statistical analyses were conducted in GraphPad Prism 10, with *p* < 0.05 considered statistically significant. The animal experiment was performed twice independently with similar results; data from one representative experiment were shown. All cell culture experiments were repeated at least three times independently.

## Results

### Pathogenic FAdV-4 infection induces mitochondrial damage and mitophagy in LMH cells

**Figure 1:**
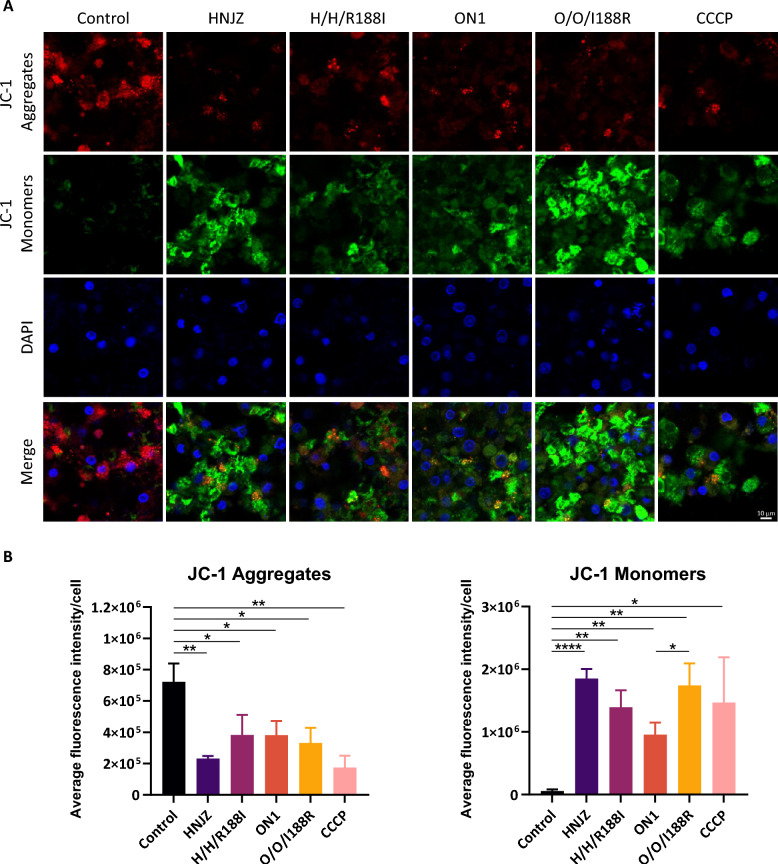
**FAdV-4-induced mitochondrial depolarization.** **A** LMH cells were infected with FAdV-4 of different virulence at a MOI of 0.001, stained with JC-1 at 48 hpi and observed using a confocal microscope. CCCP was applied as the positive control. JC-1 aggregates: red; JC-1 monomers: green; scale bar: 10 μm. In healthy mitochondria with intact membrane potential, JC-1 forms J-aggregates that exhibit intense red fluorescence with minimal green fluorescence. Following mitochondrial depolarization, JC-1 monomers predominate in the cytosol. **B** Quantification of average fluorescence intensity per cell. Data are presented as mean ± SEM (*p < 0.05, **p < 0.01, ***p < 0.001).

To investigate mitochondrial damage in LMH cells induced by FAdV-4 of different virulence, the mitochondrial membrane potential of LMH cells was assessed using the JC-1 fluorescent probe (Figure 1A). In all infected groups, significant enhancement in green fluorescence (JC-1 monomers) and reduction in red fluorescence (JC-1 aggregates) were observed, indicating decreased MMP and mitochondrial impairment caused by FAdV-4 infection (*p* < 0.05). Infection with the nonpathogenic ON1 strain resulted in a moderate accumulation of JC-1 monomers in LMH cells, suggesting relatively intact mitochondrial integrity. LMH cells infected with the attenuated H/H/R188I mutant exhibited more pronounced red fluorescence, indicating a larger amount of JC-1 aggregates in the mitochondrial matrix compared with the HNJZ-infected cells. However, the observed differences in fluorescence signals of JC-1 aggregates between the HNJZ and H/H/R188I groups were not statistically significant. In addition, as shown in Figure 1B, O/O/I188R‑infected cells exhibited a significant increase in JC‑1 monomers compared with ON1‑infected cells (*p* < 0.05). The positive control group treated with CCCP demonstrated near-complete MMP dissipation, confirming assay validity (Figure 1B).

**Figure 2:**
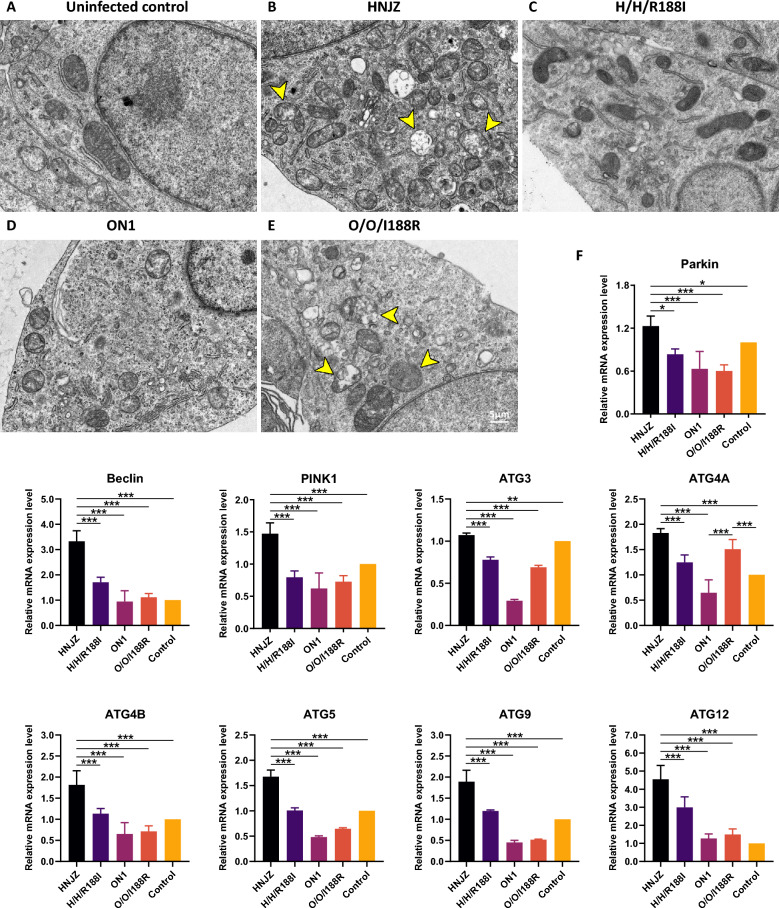
**Mitochondrial ultrastructure observation and mitophagy-related gene expression in LMH cells**. LMH cells were cultured in 6-well plates and infected with FAdV-4 strains at a MOI of 0.001. At 48 hpi, cells were harvested for TEM observation. **A**, **C**, **D** Intact mitochondria were found in uninfected, ON1-infected and H/H/R188I-infected cells. **B**, **E** Pathological features (vacuolization and cristae loss) were observed in mitochondria of HNJZ- and O/O/I188R-infected cells, as indicated by yellow arrows (× 5000). **F** Relative mRNA expression levels of mitophagy-related genes in the PINK1/Parkin pathway indicated mitophagy activation at the late infection stage in LMH cells infected with HNJZ. The expression level of ATG4A in O/O/I188R-infected cells was also elevated significantly at 72 hpi. Data are presented as mean ± SEM (*p < 0.05, **p < 0.01, ***p < 0.001).

TEM was employed to visualize mitochondrial ultrastructure. Given TEM’s stringent requirements for cellular integrity, cell samples were collected at 48 hpi to minimize cellular disintegration caused by cytopathic effects. Despite limited overall cytopathology at 48 hpi, distinct mitochondrial morphology was observed across cells infected with FAdV-4 strains of varying virulence. In the uninfected, H/H/R188I-, and ON1-infected LMH cells, mitochondria exhibited relatively intact double-membrane structures with well-defined cristae and organized matrix architecture (Figure 2A, C, D). In HNJZ- and O/O/I188R-infected cells, pronounced mitochondrial pathology was observed, including matrix vacuolization and cristae disorganization (Figure 2B, E).

To investigate whether FAdV-4 infection triggers mitophagy in vitro, mRNA expression levels of mitophagy- and autophagy-regulatory genes in LMH cells infected with different FAdV-4 strains were measured by RT-qPCR (Figure 2F). At the late stage of infection (72 hpi), HNJZ-infected LMH cells exhibited significant transcriptional activation (*p* < 0.05) of core mitophagy regulators Parkin and PINK1, as well as autophagy-regulatory genes Beclin, ATG3, ATG4A, ATG4B, ATG5, ATG9, and ATG12. This upregulation was markedly higher than that in H/H/R188I-, ON1-, and O/O/I188R-infected cells, as well as the uninfected control. Notably, the mRNA expression level of ATG4A was also significantly upregulated in O/O/I188R-infected cells (*p* < 0.001) relative to ON1-infected cells and uninfected controls, though to a lesser magnitude than that in the HNJZ group. These data demonstrated that wild-type FAdV-4 induced mitophagy via transcriptional activation of the PINK1/Parkin pathway, with Hexon residue 188 critically modulating this process. With a single residue mutation, H/H/R188I no longer induced mitochondrial damage and transcriptional activation of PINK1/Parkin-dependent mitophagy.

**Figure 3:**
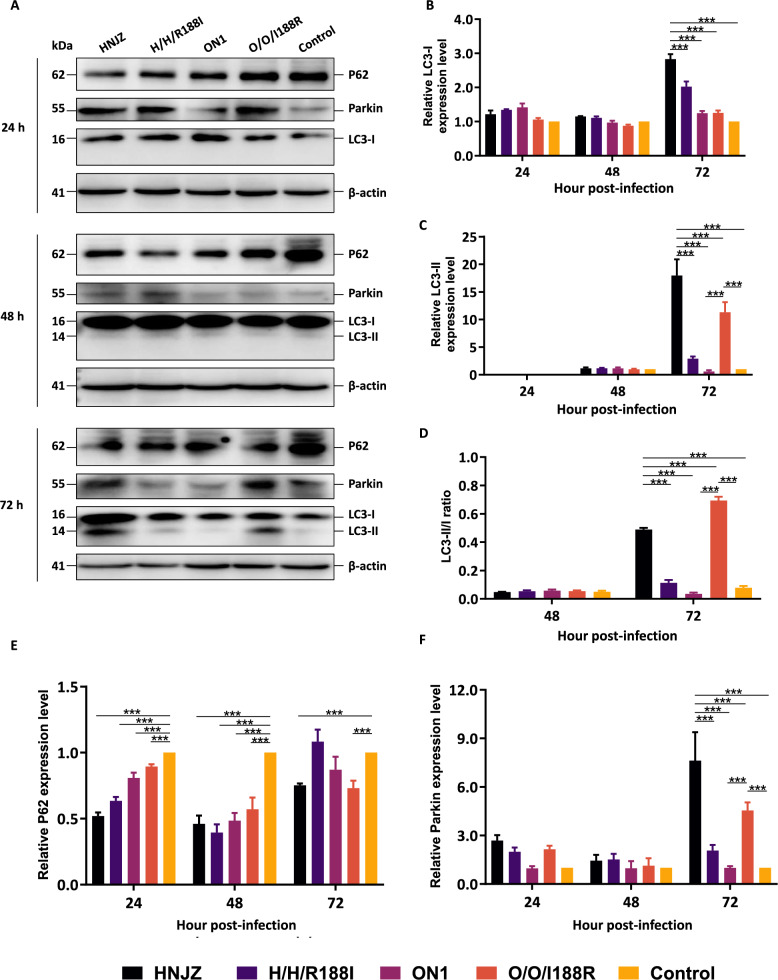
**Relative protein expression of autophagy/mitophagy-related genes in LMH cells infected with FAdV-4 of varying virulence.** **A** Representative western blots for P62, Parkin, and LC3. **B**, **C** Quantification of LC3-I and LC3-II protein expression levels. **D** The LC3-II/LC3-I ratio in HNJZ and O/O/I188R-infected LMH cells significantly increased at 72 hpi. **E**, **F** Quantification of P62 and Parkin levels. The expression of Parkin protein increased significantly at 72 hpi in HNJZ- and O/O/I188R-infected LMH cells, which aligned with the transcriptional activation of mitophagy-related genes. Data are presented as mean ± SEM (*p < 0.05, **p < 0.01, ***p < 0.001).

To further evaluate the FAdV-4-driven mitophagy, the protein expression levels of autophagy- and mitophagy-related markers LC3, P62, and Parkin in LMH cells were assessed by western blot. At 72 hpi, HNJZ-infected cells exhibited significant upregulation (*p* < 0.001) of both LC3-I and LC3-II compared with H/H/R188I-, ON1-, O/O/I188R-infected cells, and the uninfected control (Figure 3A–C). The LC3-II/LC3-I ratio, a hallmark of autophagic flux, was significantly elevated (*p* < 0.001), indicating robust autophagosome formation (Figure 3D). Similarly, O/O/I188R-infected cells showed a significantly increased LC3-II/LC3-I ratio at 72 hpi compared with the ON1-infected cells and uninfected control. All FAdV-4 strains induced downregulation (*p* < 0.001) of P62 protein at 24 and 48 hpi compared with controls (Figure 3E). By 72 hpi, HNJZ- and O/O/I188R-infected cells maintained significant P62 suppression (*p* < 0.001), whereas H/H/R188I- and ON1-infected cells showed recovery, suggesting a role for the Hexon R188I mutation in attenuating autophagic flux. Parkin expression in HNJZ-infected cells was significantly elevated at 72 hpi versus all other groups (*p* < 0.001). O/O/I188R infection also induced significant Parkin upregulation (*p* < 0.001) compared with ON1-infected and control cells, though less pronounced than HNJZ (Figure 3F).

**Figure 4:**
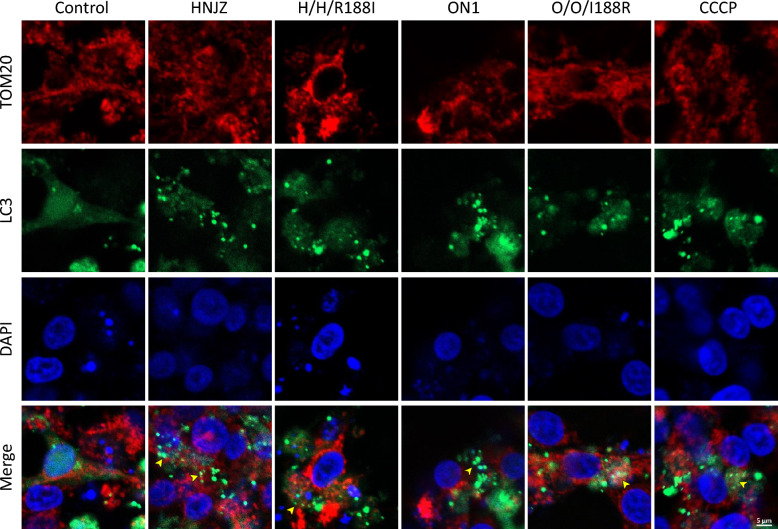
**Colocalization of LC3 and TOM20 in LMH cells infected with FAdV-4 strains of varying virulence.**

Immunofluorescence staining was used to detect the co-localization of TOM20 and LC3. LMH cells were transfected with a pEGFP-LC3 plasmid, infected with FAdV-4 of different virulence, and immunostained for TOM20. LC3-positive autophagosomes were observed in all FAdV-4-infected groups (Figure 4). Enhanced colocalization of LC3 and TOM20 was found in the HNJZ- and O/O/I188R-infected cells.

### Pathogenic FAdV-4 infection induces mitochondrial damage and mitophagy in hepatocytes in vivo

**Figure 5:**
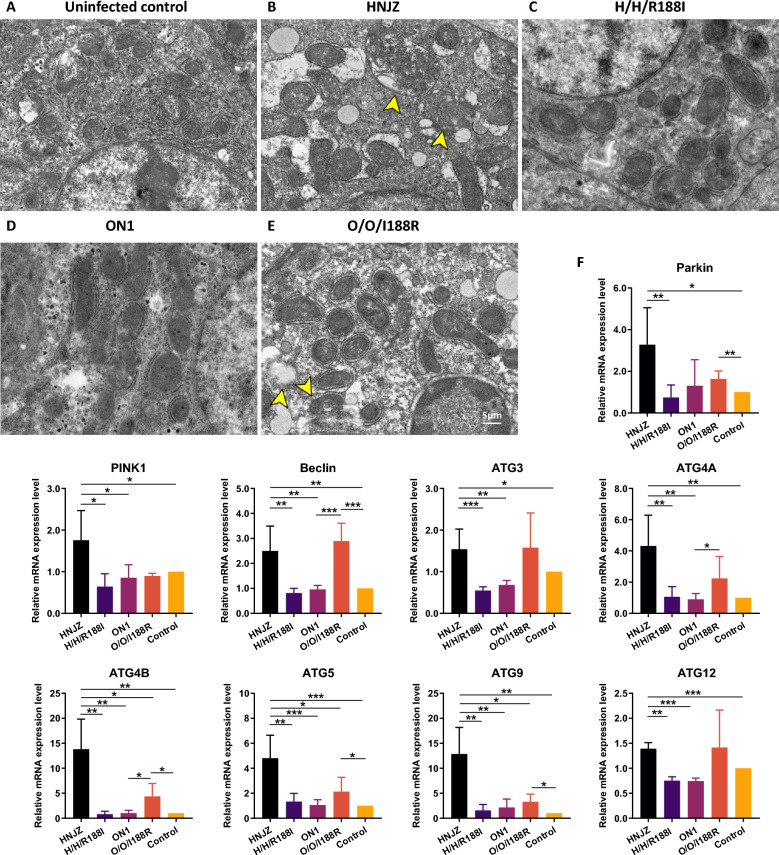
**Mitochondrial ultrastructure observation and mitophagy-related gene expression in hepatocytes.** **A** SPF chickens were infected with FAdV-4 of varying virulence. Chickens were euthanized at 48 hpi to collect fresh liver tissue samples for TEM examination. Normal mitochondrial morphology was observed in uninfected hepatocytes with a round shape and intact matrix. **B** Severe mitochondrial damage was found in HNJZ-infected hepatocytes, as indicated by yellow arrows. **C**, **D**, **E** Subcritical pathological changes of mitochondria were detected in the ON1-, H/H/R188I-, and O/O/I188R-infected hepatocytes (× 5000). **F** Relative mRNA expression levels of mitophagy related genes in hepatocytes indicated mitophagy activation in chickens infected with HNJZ and O/O/I188R. Data are presented as mean ± SEM (*p < 0.05, **p < 0.01, ***p < 0.001).

To validate mitochondrial injury induced by FAdV-4 infection in vivo, SPF chickens were infected with FAdV-4 strains of varying virulence. Liver tissue samples were collected for TEM. The hepatocytes from the uninfected control showed complete mitochondrial ultrastructure with regular spherical shapes and densely packed cristae (Figure 5A). Severe mitochondrial pathology was observed in HNJZ-infected chickens, with swelling and deformation of mitochondria, loss of structural integrity, and membrane rupture (Figure 5B). In the ON1-, H/H/R188I-, and O/O/I188R-infected chickens, the mitochondria retained overall structural integrity but displayed localized swelling and cristae disorganization, suggesting milder damage (Figure 5C–E).

The mRNA expression levels of mitophagy- and autophagy-related genes in hepatocytes were quantified by RT-qPCR. In the HNJZ-infected group, significant upregulation of Parkin was observed compared with H/H/R188I-infected and the control groups (*p* < 0.05); significant elevations of PINK1, Beclin, ATG3, ATG4A, and ATG12 were detected compared with H/H/R188I-, ON1-infected, and the uninfected control groups (*p* < 0.05); significantly increased mRNA expressions of ATG4B, ATG5, and ATG9 were observed relative to all other groups (*p* < 0.05). In the O/O/I188R-infected group, the expressions of Parkin, ATG5, and ATG9 were significantly upregulated (*p* < 0.05) versus the control; the expressions of Beclin and ATG4B were significantly elevated (*p* < 0.05) compared with ON1-infected and control groups (Figure 5F).

**Figure 6:**
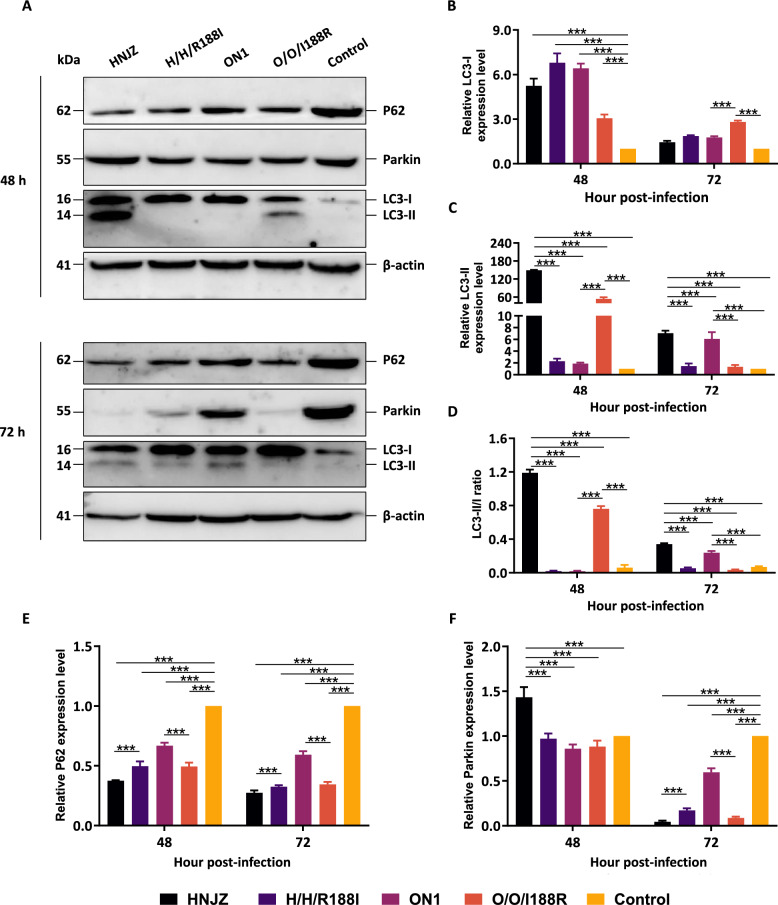
**Relative protein expression of autophagy/mitophagy-related genes in chicken hepatocytes infected with FAdV-4 of varying virulence.** **A** Representative western blots for P62, Parkin and LC3. **B**, **C** Quantification of LC3-I and LC3-II protein expression levels. **D** LC3-II/LC3-I ratio. **E**, **F** Quantification of P62 and Parkin levels. The LC3-II/LC3-I ratio and Parkin protein expression indicated that mitophagy was activated in HNJZ- and O/O/I188R-infected chickens at 48 hpi and declined at 72 hpi. Data are presented as mean ± SEM (*p < 0.05, **p < 0.01, ***p < 0.001).

To further confirm FAdV-4-induced mitophagy in hepatocytes, the protein expression levels of LC3, P62, and Parkin were examined via western blot (Figure 6A). At 48 hpi, all infected groups exhibited marked upregulation of LC3-I compared with the uninfected control (*p* < 0.001). As shown in Figure 6C, HNJZ‑infected hepatocytes exhibited significantly higher LC3‑II levels than all other groups (*p* < 0.001). O/O/I188R‑infected hepatocytes also displayed significantly increased LC3‑II levels compared with the ON1‑infected and control groups (*p* < 0.001). Accordingly, the LC3-II/LC3-I ratio was significantly elevated in HNJZ-infected hepatocytes relative to all other groups (*p* < 0.001). A similar but less pronounced increase in the LC3-II/LC3-I ratio was observed in O/O/I188R-infected cells compared with ON1-infected and control groups (*p* < 0.001) (Figure 6D).

At 72 hpi, only O/O/I188R-infected cells maintained significantly upregulated LC3-I expression compared with the ON1-infected and the control groups (*p* < 0.001) (Figure 6B). Notably, LC3-II protein levels remained high in HNJZ-infected hepatocytes and, interestingly, also increased in ON1-infected cells at this time point. Consequently, the LC3-II/LC3-I ratio in the HNJZ group remained significantly higher than in all other groups (*p* < 0.001). Furthermore, the ON1-infected group exhibited a significantly higher LC3-II/LC3-I ratio compared with the O/O/I188R-infected and control groups (*p* < 0.001). This late-stage increase in the ON1 group likely results from impaired autophagic flux rather than enhanced autophagic induction.

The protein expression level of P62 in the uninfected control group was significantly higher than in all FAdV-4-infected groups at 48 and 72 hpi (*p* < 0.001) (Figure 6E). Strain-specific comparisons indicated that the expression of P62 was significantly higher in H/H/R188I- and ON1-infected hepatocytes than in the HNJZ- and O/O/I188R-infected groups, accordingly. These results indicate enhanced autophagic flux in HNJZ- and O/O/I188R-infected hepatocytes, as P62 degradation is a hallmark of autophagosome-lysosome fusion and substrate clearance. At 48 hpi, Parkin protein expression in HNJZ-infected hepatocytes was significantly upregulated (*p* < 0.001) compared with all other groups (H/H/R188I, ON1, O/O/I188R, and controls), indicating robust mitophagy initiation. However, at 72 hpi, a global downregulation of Parkin in all FAdV-4-infected groups was observed versus the uninfected control (Figure 6F). Parkin expression was significantly reduced in HNJZ-infected hepatocytes versus H/H/R188I-infected hepatocytes (*p* < 0.001), and in O/O/I188R-infected hepatocytes versus ON1-infected hepatocytes (*p* < 0.001). This temporal shift suggested mitophagy inhibition in the pathogenic FAdV-4-infected groups at late infection stages.

### Pathogenic FAdV-4 infection induces mitochondrial damage in cardiomyocytes in vivo

**Figure 7:**
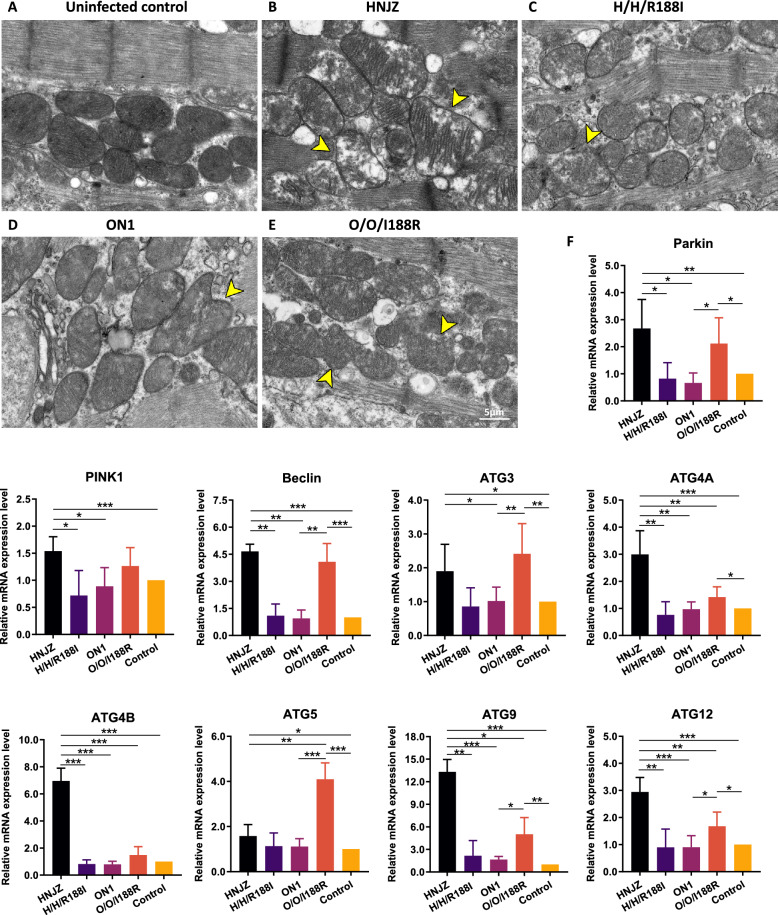
**Mitochondrial ultrastructure observation and mitophagy-related gene expression in cardiomyocytes.** **A** Densely packed, healthy mitochondria were observed in uninfected cardiomyocytes. **B**, **E** Severe mitochondrial injuries were found in HNJZ- and O/O/I188R-infected cardiomyocytes. **C**, **D** Mild pathological changes of mitochondria were detected in the ON1- and H/H/R188I-infected cardiomyocytes (× 8000). **F** Relative mRNA expression levels of mitophagy-related genes in cardiomyocytes of chickens infected with FAdV-4 strains of different virulence. Data are presented as mean ± SEM (*p < 0.05, **p < 0.01, ***p < 0.001).

The impact of FAdV-4 infection on mitochondrial integrity in cardiomyocytes in vivo was evaluated by TEM. Under normal conditions, mitochondria in cardiomyocytes exhibited regular elliptical morphology, intact double-membrane structures, and well-organized cristae (Figure 7A). The mitochondria in HNJZ- and O/O/I188R-infected cardiomyocytes exhibited severe damage including swelling and deformation, loss of structural integrity, membrane rupture, cristae disorganization, and reduced mitochondrial number (Figure 7B, E). In ON1- and H/H/R188I-infected cardiomyocytes, mitochondria also displayed swelling and focal cristae remodeling, indicating mild damage (Figure 7C, D).

At the transcriptional level, cardiomyocytes infected with the pathogenic HNJZ strain exhibited significant upregulation of mitophagy-related genes, including Parkin, PINK1, and Beclin, compared with the H/H/R188I-infected, ON1-infected, and control groups (*p* < 0.05); significantly increased mRNA expressions of ATG4A/4B, ATG9, and ATG12 were detected relative to all other groups (*p* < 0.05). Significant elevations in Parkin, Beclin, ATG3, ATG5, ATG9, and ATG12 mRNA levels were observed in O/O/I188R-infected cells compared with the ON1-infected and control groups (*p* < 0.05) (Figure 7F), indicating activation of autophagosome biogenesis.

**Figure 8:**
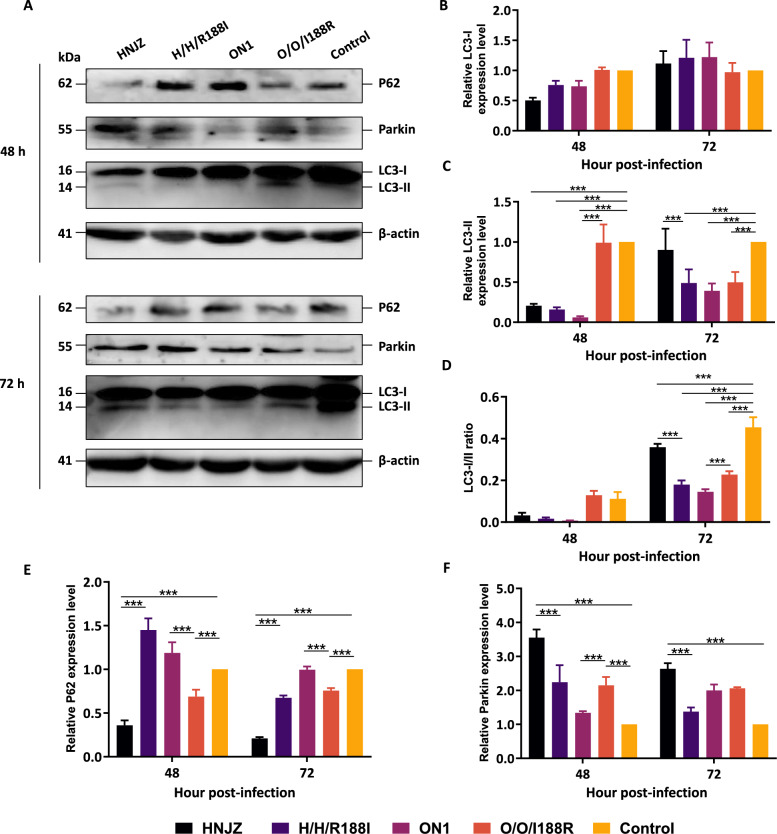
**Relative protein expression of autophagy/mitophagy-related genes in chicken cardiomyocytes infected with FAdV-4 of varying pathogenicity.**

Evaluation of total protein expression of LC3 indicated a global reduction in FAdV-4-infected cardiomyocytes compared with the uninfected control (Figure 8A–C). However, a time-dependent increase in LC3-II/LC3-I ratio was observed, with HNJZ- and O/O/I188R-infected groups showing significant elevation at 72 hpi versus H/H/R188I and ON1 groups (*p* < 0.001) (Figure 8D). The expression level of P62 protein in HNJZ-infected cardiomyocytes was significantly downregulated at 48 and 72 hpi compared with H/H/R188I-infected and control groups (*p* < 0.001); similarly, P62 suppression was also observed in O/O/I188R-infected cells versus ON1-infected and control groups (*p* < 0.001) (Figure 8E). Parkin expression in HNJZ-infected cardiomyocytes was significantly elevated (*p* < 0.001) at 48 and 72 hpi compared with H/H/R188I-infected and control groups. Significantly upregulated Parkin expression was also observed in O/O/I188R-infected cardiomyocytes with respect to ON1-infected group and the control group at 48 hpi (Figure 8F).

### Inhibiting mitophagy suppressed FAdV-4 replication

**Figure 9:**
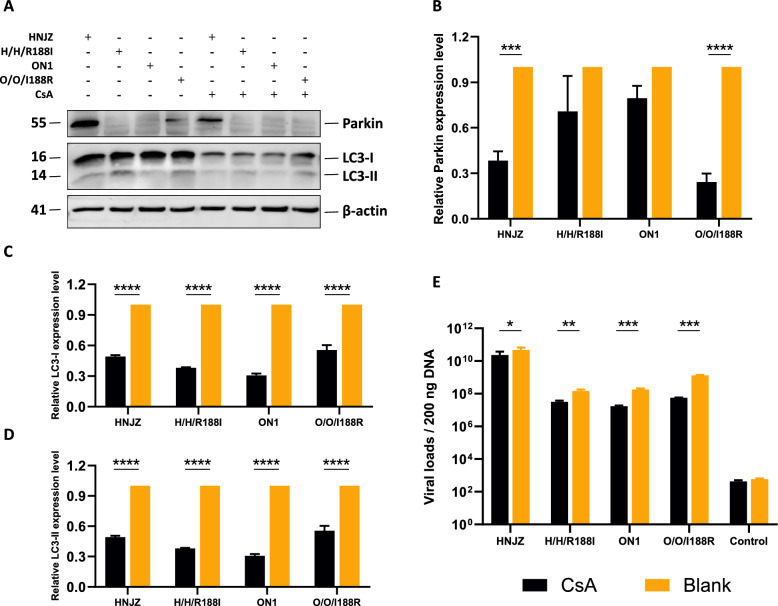
**Inhibiting mitophagy by CsA suppressed FAdV-4 replication.** LMH cells were treated with CsA at 1 μM for 6 h and then infected with FAdV-4 strains of different virulence. **A** The protein expression of LC3 and Parkin in LMH cells under different treatment conditions. **B** Greyscale analysis of Parkin protein expression. **C**, **D** Quantification of LC3 expression. **E** FAdV-4 viral loads detected by RT-qPCR. Data are presented as mean ± SEM (*p < 0.05, **p < 0.01, ***p < 0.001).

**Figure 10:**
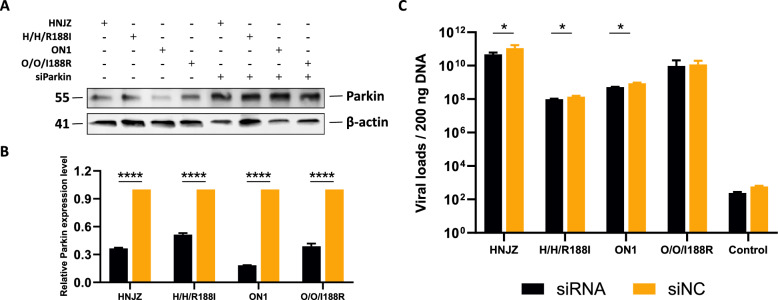
**Inhibiting mitophagy by siParkin suppressed FAdV-4 replication.** LMH cells were transfected with siParkin prior to FAdV-4 infection. **A** The protein expression of Parkin in LMH cells under different treatment conditions. **B** Quantification of Parkin expression. **C** FAdV-4 viral loads detected by RT-qPCR. Data are presented as mean ± SEM (*p < 0.05, **p < 0.01, ***p < 0.001).

To investigate the role of mitophagy in FAdV-4 replication, LMH cells were pre-treated with 1 μM CsA for 6 h prior to viral infection. Western blot analysis revealed a marked suppression of mitophagy, evidenced by significantly reduced expression of LC3-I, LC3-II, and Parkin in CsA-treated cells (*p* < 0.001) (Figure 9A–D). Strikingly, all FAdV-4 strains exhibited restricted replication in CsA-treated cells compared with the untreated infected controls, with an average tenfold reduction in viral loads (*p* < 0.01) (Figure 9E). To further identify the influence of mitophagy on FAdV-4 replication, the expression of Parkin was suppressed by transfection with Parkin siRNA (Figure 10A, B). The viral loads of FAdV-4 in the transfected LMH cells were significantly reduced in the HNJZ-, H/H/R188I-, and ON1-infected groups (*p* < 0.01) (Figure 10C).

## Discussion

Accumulating data highlight the dual role of mitophagy in viral infections. Mitochondrial ROS (mtROS) activate inflammatory cascades, contributing to organ damage. For instance, mtROS trigger NLRP3 inflammasome activation, promoting IL-1β and IL-18 maturation [26]. While mitophagy mitigates mtROS accumulation and sustains mitochondrial function, dysregulated or excessive autophagy may exacerbate pathology. Avian reovirus (ARV) infection induces autophagic vesicles containing mitochondria in chicken cardiac and lymphoid tissues, leveraging autophagy to amplify viral replication and IL-1β production [27]. Hepatitis C virus (HCV) upregulates Parkin expression in infected hepatocytes, promoting mitophagy to sustain viral persistence; silencing PINK1/Parkin inhibits HCV replication [28, 29]. Porcine reproductive and respiratory syndrome virus (PRRSV) stimulates mitophagy to suppress apoptosis, facilitating viral propagation [30]. Similarly, pseudorabies virus infection triggers PINK1-Parkin-mediated mitophagy and suppresses IFN-I production, thereby promoting viral replication [31]. These findings underscore mitophagy as a critical interface between mitochondrial homeostasis, inflammation, and viral pathogenesis. Building upon established evidence that Hexon residue 188 is a critical determinant of FAdV-4 pathogenicity [8, 9], this study aims to systematically characterize FAdV-4-induced mitochondrial damage and associated mitophagy in vitro and in vivo, and to evaluate the potential role of Hexon residue 188 in modulating these processes.

The study utilized TEM to observe mitochondrial damage in LMH cells and in cardiomyocytes and hepatocytes from chickens infected with HNJZ, ON1, H/H/R188I, O/O/I188R strains, as well as uninfected controls. The ultrastructure of mitochondria in LMH cells, hepatocytes and cardiomyocytes infected with HNJZ and O/O/I188R exhibited mitochondrial swelling, membrane rupture, and cristae disorganization, indicating direct mitochondrial injury induced by pathogenic FAdV-4 infection. The mitochondrial impairment caused by FAdV-4 infection was also evidenced by the reduced MMP. Subsequently, RT-qPCR and western blot were employed to detect the expression of autophagy and mitophagy-related genes, including Parkin, PINK1, LC3, P62, Beclin, and ATGs. The pronounced mRNA upregulation of PINK1/Parkin and autophagy-related genes in HNJZ-infected groups indicated transcriptional activation of mitophagy in LMH cells, hepatocytes and cardiomyocytes by pathogenic FAdV-4 infection. Elevated expression of ATG4A/4B, the critical enzymes for LC3 lipidation, together with the observed PINK1/Parkin upregulation and enhanced colocalization of LC3 and TOM20, implied enhanced autophagosome biogenesis in response to mitochondrial damage.

The marked LC3-II/LC3-I ratio elevation, suppression of P62 and upregulation of Parkin proteins in HNJZ and O/O/I188R-infected LMH cells and chicken hepatocytes implied autophagosome formation, driven by severe mitochondrial damage. Both LC3-II/LC3-I ratio and Parkin protein expression in HNJZ-infected hepatocytes decreased dramatically at 72 hpi in vivo compared with 48 hpi, indicating the time-dependent nature of FAdV-4-mediated mitophagy. At the early stage of FAdV-4 infection, the pathogenic strain HNJZ activated mitophagy to manage acute mitochondrial damage. During late infection, viral persistence suppressed Parkin expression, disrupting mitochondrial quality control and exacerbating tissue injury.

In chicken cardiomyocytes, Parkin protein upregulation and p62 degradation in the pathogenic HNJZ and O/O/I188R-infected groups at 48 hpi aligned with the acute mitochondrial damage observed in TEM, facilitating the ubiquitination and clearance of damaged mitochondria. However, the protein expression of LC3-I and LC3-II in the infected groups was significantly lower compared with the control group. This could be caused by the rapid and severe mitochondrial damage and mitophagy induced by massive viral infection, leading to rapid degradation of LC3 that exceeded its synthesis.

Our previous findings established that the Hexon residue 188 mutation attenuated the virulence of the pathogenic FAdV-4 strain HNJZ while enhancing that of the nonpathogenic ON1 strain [6]. The present study further identified Hexon residue 188 as a central regulator of FAdV-4 pathogenicity through its role in modulating mitophagy. Mitophagy in cells infected with the recombinant H/H/R188I strain carrying the Hexon residue 188 mutation was significantly reduced compared with the parental pathogenic HNJZ strain. Conversely, mitophagy in cells infected with the O/O/I188R strain was markedly enhanced relative to the parental non-pathogenic ON1 strain. Transcriptional analysis revealed that the O/O/I188R mutant partially phenocopied HNJZ in the mitophagy-regulatory gene expression profile, suggesting that the I188R substitution contributed to mitochondrial dysregulation. The Hexon R188I mutation in HNJZ attenuated these effects, consistent with its reduced pathogenicity, underscoring the functional significance of Hexon residue 188 in mitophagy and viral pathogenicity.

In our previous study, the I188R mutation introduced into the nonpathogenic ON1 background (O/O/I188R) enhanced viral pathogenicity and induced mild clinical signs including depression and reduced feed intake, but did not cause mortality in infected chickens [8]. This observation, when combined with the present data, suggests that while Hexon residue 188 is a critical regulator of FAdV-4-induced mitophagy, additional viral genes or host factors are still required for the full lethal phenotype associated with the highly pathogenic HNJZ strain. The fact that O/O/I188R activates mitophagy to a level comparable to that of HNJZ yet fails to cause death implies that other virulence determinants (e.g., additional mutations in HNJZ outside Hexon) contribute to the lethal outcome. This is consistent with our earlier work demonstrating that multiple genes, including Fiber‑2, are also involved in FAdV‑4 pathogenicity [10]. Future studies should explore potential synergistic interactions between Hexon residue 188 and other viral proteins in driving mortality in HHS.

To elucidate the role of mitophagy in FAdV-4 replication, LMH cells were pretreated with CsA, a specific inhibitor of mitochondrial permeability transition pore (mPTP) formation that blocks Parkin-mediated mitophagy by inhibiting cyclophilin D [24], prior to viral infection. Western blot analysis demonstrated marked suppression of mitophagic flux, as evidenced by significantly reduced expression of LC3-I, LC3-II and Parkin. This suppression coincided with reduced viral replication across all FAdV-4 strains at 48 hpi, particularly in variants H/H/R188I, ON1, and O/O/I188R. To further validate these findings, LMH cells were transfected with siParkin to suppress Parkin protein expression, and viral replication was reassessed. Consistent with the CsA treatment results, Parkin knockdown significantly suppressed FAdV-4 replication in HNJZ-, H/H/R188I-, and ON1-infected cells. Collectively, our study demonstrated through both pharmacological inhibition (CsA) and genetic intervention (siParkin) that mitophagy played a proviral role in FAdV-4 replication. These findings revealed a novel mechanism whereby FAdV-4 hijacked host mitophagy to enhance viral replication efficiency during early infection stages.

Viruses have evolved diverse strategies to manipulate host mitophagy pathways, ultimately facilitating their own replication through distinct mechanisms. Previous studies have shown that FAdV‑4 infection induces general autophagy in chicken hepatocytes, a nonselective process triggered by various cellular stresses, including viral infection. By contrast, the present study specifically identifies mitophagy, a selective form of autophagy that targets damaged mitochondria via the PINK1/Parkin pathway. While general autophagy occurs during FAdV‑4 infection, our work reveals that the virus specifically hijacks the mitophagy machinery, a more targeted and functionally significant mechanism, to promote its replication. However, the precise mechanism through which Hexon residue 188 influences this process requires further investigation. We hypothesize that this structural viral protein might interact with host transcription factors such as NF-κB, known regulators of mitophagy-related genes [26], thereby modulating the PINK1/Parkin pathway. Future studies are required to investigate the direct or indirect molecular interactions between Hexon and the mitophagy machinery, which will further clarify how FAdV-4 hijacks host cellular processes to promote its replication.

A limitation of this study is that, owing to the lack of commercially available antibodies that cross-react with chicken orthologs, the protein expression of several autophagy-related factors (e.g., ATGs) could not be examined. Nevertheless, the messenger RNA (mRNA) changes, together with validated protein data for LC3, P62, and Parkin, support the conclusions. In addition, the animal study had a relatively small sample size in each chicken group, which might constrain the statistical power of our findings and their generalizability to broader populations.

The findings of this study highlighted Hexon residue 188 as a promising therapeutic target. Its targeted modification may facilitate the development of attenuated live vaccines and vectored vaccines against FAdV-4 through deliberate alteration of virus-mitochondria interactions [32, 33]. Moreover, our in vitro data suggest that pharmacological or genetic inhibition of mitophagy suppresses viral replication, raising the possibility that targeting the mitophagy pathway itself may have therapeutic value. Future in vivo studies using CsA or Parkin knockdown in chickens are warranted to evaluate the translational potential of this strategy. Together, these molecular insights provide a foundation for novel antiviral approaches that disrupt the viral hijacking of host mitochondrial quality control pathways.

## Data Availability

The datasets generated and/or analyzed during the current study are available in the ScienceDB repository (10.57760/sciencedb.32333).
